# Identification of ubiquinol cytochrome *c* reductase hinge (UQCRH) as a potential diagnostic biomarker for lung adenocarcinoma

**DOI:** 10.1098/rsob.150256

**Published:** 2016-06-29

**Authors:** Feng Gao, Qicai Liu, Guoping Li, Feng Dong, Minglian Qiu, Xiaoting Lv, Sheng Zhang, Zheng Guo

**Affiliations:** 1 Department of Pathology, Fujian Medical University, Fuzhou 350005, People's Republic of China; 2 Department of Laboratory Medicine, Fujian Medical University, Fuzhou 350005, People's Republic of China; 3 Department of Radiation Oncology, Fujian Medical University, Fuzhou 350005, People's Republic of China; 4 Department of Chest Surgery, Fujian Medical University, Fuzhou 350005, People's Republic of China; 5 Department of Respiratory, the First Affiliated Hospital, Fujian Medical University, Fuzhou 350005, People's Republic of China; 6 Department of Bioinformatics, Fujian Medical University, Fuzhou 350005, People's Republic of China

**Keywords:** lung adenocarcinoma, ubiquinol-cytochrome *c* reductase hinge, serum biomarker, mitochondrial membrane potential

## Abstract

Ubiquinol cytochrome *c* reductase hinge (UQCRH) is a novel protein that localizes in the mitochondrial membrane and induces mitochondrial reactive oxygen species (ROS) generation. It had a high expression rate of 87.10% (108/124) in lung adenocarcinoma. Moreover, serum UQCRH level in patients with lung adenocarcinoma was significantly increased compared with that of pneumonia patients (*p* < 0.0001) and normal control subjects (*p* < 0.0001). Receiver operating characteristic curve analysis using an optimal cut-off value of 162.65 pg ml^−1^ revealed sensitivity and specificity for the diagnosis of lung adenocarcinoma of 88.7% and 85.7%, respectively, with an area under the curve of 0.927 (95% CI: 0.892 to 0.962, *p* < 0.0001). Serum UQCRH discriminates lung adenocarcinoma patients from the population without cancer with considerable sensitivity and specificity, but it does not distinguish between heavy smokers and lung adenocarcinoma patients. Serum UQCRH could be a potential diagnostic biomarker for lung adenocarcinoma.

## 1. Introduction

Lung cancer is the most common cause of global cancer-related mortality, leading to over a million deaths each year, and adenocarcinoma is the most common histological type, accounting for approximately one-half of lung cancer cases. It is frequently diagnosed at advanced stages, resulting in a 5-year survival rate of only 16%; early diagnosis is essential to reduce mortality of this fatal disease [1,2]. A variety of invasive and non-invasive techniques for the early detection of lung cancer have been studied, including low-dose chest computed tomography (LDCT) screening for high-risk patients, but this concerns exposure to radiation and a high false-positive rate. Serum or plasma is the preferred choice for the development of candidate biomarkers because of minimal invasiveness and easy accessibility. However, no biomarker of lung adenocarcinoma is available for use in clinical practice owing to insufficient evidence of their diagnostic specificity, despite numerous tumour-specific proteins or molecules in blood such as carcinoembryonic antigen (CEA), neurone specific enolase (NSE) and CYFRA21-1, having been shown to be potential biomarkers [2,3]. The sensitivity of lung cancer diagnosis does not reach 50% even with a combination of these markers [4,5].

Genetic instability and cellular energy metabolism changes are two important features of cancer. Genetic instability makes it possible for cancer cells to proliferate abnormally, and energy metabolism changes dominated by mitochondria are the driving force for tumour cell proliferation [6]. Proteins identified by analysing breast cancer cell (MCF7) nucleus and mitochondria proteomics, such as UQCRH, UQRC2, NDUFA5, etc., have been proved to participate in nuclear/mitochondrial RNA translation in glycolysis and oxidative phosphorylation, which is one of the important ways in which mitochondria are involved in cancer [6–8]. Reactive oxygen species (ROS) are required for normal cellular homeostasis and physiology in several subcellular events, such as enzyme activation, signal transduction and gene expression [8]. When pro-oxidant/anti-oxidant equilibrium is lost, oxidative stress is generated, damaging intracellular molecules including DNA [9]. The damaged DNA usually results in cell death, but it could also bring about carcinogenesis in the case of a concomitant impaired DNA repair mechanism. However, the exact mechanism of intracellular ROS production is not clearly understood. Ubiquinol-cytochrome *c* reductase hinge (UQCRH) is a novel protein located in the mitochondrial membrane which is reported to regulate the intracellular production of ROS [10,11]. We performed this study to investigate whether serum UQCRH increased in patients with lung adenocarcinoma compared with the population without lung cancer. Furthermore, we assessed the clinical parameters that may be related to UQCRH expression and evaluated the potential diagnostic effectiveness of serum UQCRH for lung adenocarcinoma.

## 2. Results

### 2.1. Patient characteristics and ubiquinol cytochrome *c* reductase hinge protein expression in lung cancer patients

A total of 124 lung adenocarcinoma patients were enrolled in the study. The patient characteristics are shown in table 1. The pre-operative serum UQCRH level in male patients (*n* = 70, 290.55 ± 93.36 pg ml^−1^) was significantly higher than that in female patients (*n* = 54, 207.36 ± 70.89 pg ml^−1^, *p* < 0.0001); the median serum UQCRH from the patients who smoked (more than 500 cigarettes per year; *n* = 45, 332.42 pg ml^−1^) was significantly higher than that of the mild smoking group (*n* = 15, 231.22 pg ml^−1^, *p* < 0.0001) and that of the non-smoking group (*n* = 64, 199.22 pg ml^−1^, *p* = 0.0009). There was also a difference between the degree of differentiation of the lung adenocarcinoma: serum UQCRH level in the patients with poorly differentiated adenocarcinoma (*n* = 55, 326.45 ± 90.08 pg ml^−1^) was higher than that in the highly differentiated adenocarcinoma group (*n* = 69, 196.83 ± 43.11 pg ml^−1^, *p* < 0.0001). It is worth noting that smoking is significantly related to tumour stage in patients with lung adenocarcinoma, and heavy smokers are more likely to get lung cancer and also have higher UQCRH to begin with, so the assay will be unable to distinguish the effects of smoking from the presence of cancer (table 2).

**Table 1.** Patient characteristics of epidemiology study, stratified by gender, smoking history, histology and stage.

variable	cases	serum concentration (pg ml^−1^)	*p*-value
sex			0.016
male	70	290.55 ± 93.36	
female	54	207.36 ± 70.89	
history of smoking			
never-smoker	64	199.22 (25–75%,168.93–238.38)	<0.0001
ever-smoker (cigarettes per year)			
<500	15	231.22 (25–75%,172.93–260.72)	0.0009
≥500	45	332.42 (25–75%,258.84–415.85)	
age (years)			0.4648
<60	34	244.19 (25–75%,197.06–290.61)	
≥60	90	230.65 (25–75%,173.65–296.19)	
tumour stage			<0.0001
I or II	69	196.83 ± 43.11	
III or IV	55	326.45 ± 90.08

**Table 2.** The relationship between smoking and tumour stage in patients with lung adenocarcinoma. *p* = 6.097184056415236 × 10^−11^.

	tumour stage I or II, *n*	tumour stage III or IV, *n*	*χ*^2^	*p*
smoking status			47.04	<0.0001
never-smoker	52	12		
<500	10	5		
≥500	7	38		

### 2.2. Serum ubiquinol cytochrome *c* reductase hinge content detection

**Figure 1.**
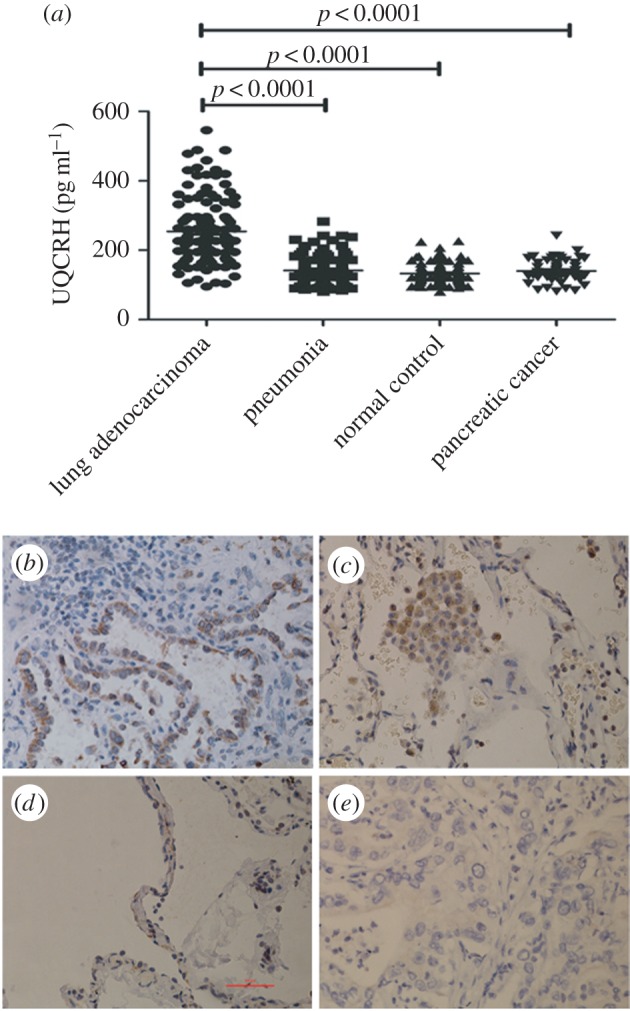
The expression of serum UQCRH in lung adenocarcinoma. (*a*) Densitometry analysis results show the serum UQCRH in lung adenocarcinoma is significantly increased compared with in non-tumour tissue. Bars denote median and interquartile range. (*b*) UQCRH-positive tumour tissues. The cells with cytoplasmic staining were clustered in most regions of the tumour tissues. The ratio of the stained cells in these areas was more than 35%. (*c*) UQCRH-medium positive tissue from samples adjacent to lung adenocarcinoma. The cytoplasm of cancer cells was stained in a scattered pattern. The ratio of the positive cells was 18–35%. (*d*) UQCRH-negative normal lung tissues or pulmonary bullae. The staining of cytoplasm was not detected in epithelial or interstitial tissue. (*e*) UQCRH-low positive pancreatic tumour tissues.

As shown in figure 1*a*, serum UQCRH of patients with lung adenocarcinoma (*n* = 124, 254.32 ± 93.65 pg ml^−1^) was significantly higher than that of pneumonia (*n* = 90, 141.92 ± 43.54 pg ml^−1^, *p* < 0.0001) and pancreatic cancer patients (*n* = 53, 140.04 ± 32.28 pg ml^−1^, *p* < 0.0001) and normal control subjects (*n* = 119, 132.56 ± 27.25 pg ml^−1^, *p* < 0.0001).

### 2.3. Immunohistochemical expression of ubiquinol cytochrome *c* reductase hinge in lung adenocarcinoma tissues

In tumour tissues the rate of UQCRH positive cells (high cytoplasmic expression) was as high as 87.10% (108/124) (figure 1*b*) and in pericancerous tissue was 38.71% (48/124) (figure 1*c*). However, in normal lung tissues the rate of UQCRH positive cells was less than 5% (1/22) (figure 1*d*), and UQCRH expression in pancreatic cancer tissue was low (figure 1*e*).

### 2.4. Ubiquinol cytochrome *c* reductase hinge mRNA expression studies in lung tumour

**Figure 2.**
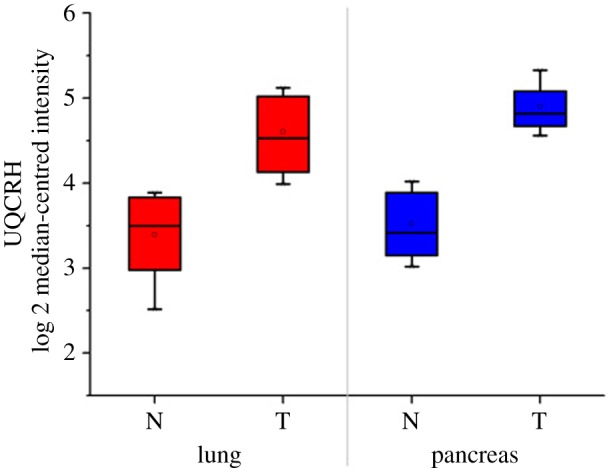
Increased UQCRH mRNA expression in lung adenocarcinoma. UQCRH mRNA of lung adenocarcinoma (T) is 1.31-fold that in normal tissue (N) and UQCRH mRNA of pancreatic cancer tissue is 1.40-fold that in normal pancreatic tissue.

Tissues from six cases of lung adenocarcinoma, six cases of pancreatic cancer and normal tissues away from the tumours were analysed for UQCRH expression. Real time PCR results (figure 2) indicated that UQCRH mRNA of lung adenocarcinoma is 1.31-fold that of normal tissue, and UQCRH mRNA of pancreatic cancer tissue is 1.40-fold that of normal pancreatic tissue.

### 2.5. The effect of ubiquinol cytochrome *c* reductase hinge on the mitochondrial membrane potential

**Figure 3.**
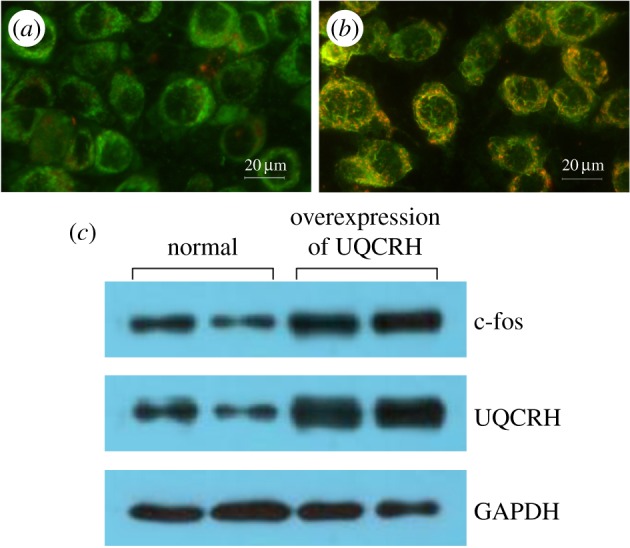
Overexpression of UQCRH in A549 cells. (*a*) Mitochondrial membrane potential in A549 cells with normal expression (original magnification ×400); (*b*) mitochondrial membrane potential in A549 cells with overexpression of UQCRH (original magnification ×400); (*c*) UQCRH induced high expression of c-fos.

To measure the collapse of electrochemical gradient across the mitochondrial membrane, we stained cells with JC-1 dye that aggregates in healthy mitochondria and fluoresces red. By using an inverted fluorescence microscope, we observed the mitochondrial membrane potential of A549 cells (red light); the cells exposed to high UQCRH (UQCRH overexpression) exhibited an increase in JC-1 staining compared with the untreated control cells (figure 3*a,b*). In order to further demonstrate the effect of UQCRH on A549 cells, we included the results of western blotting. This showed that UQCRH induced high c-fos expression, which indicated that UQCRH can promote tumour progression.

### 2.6. Diagnostic value of serum ubiquinol cytochrome *c* reductase hinge

**Figure 4.**
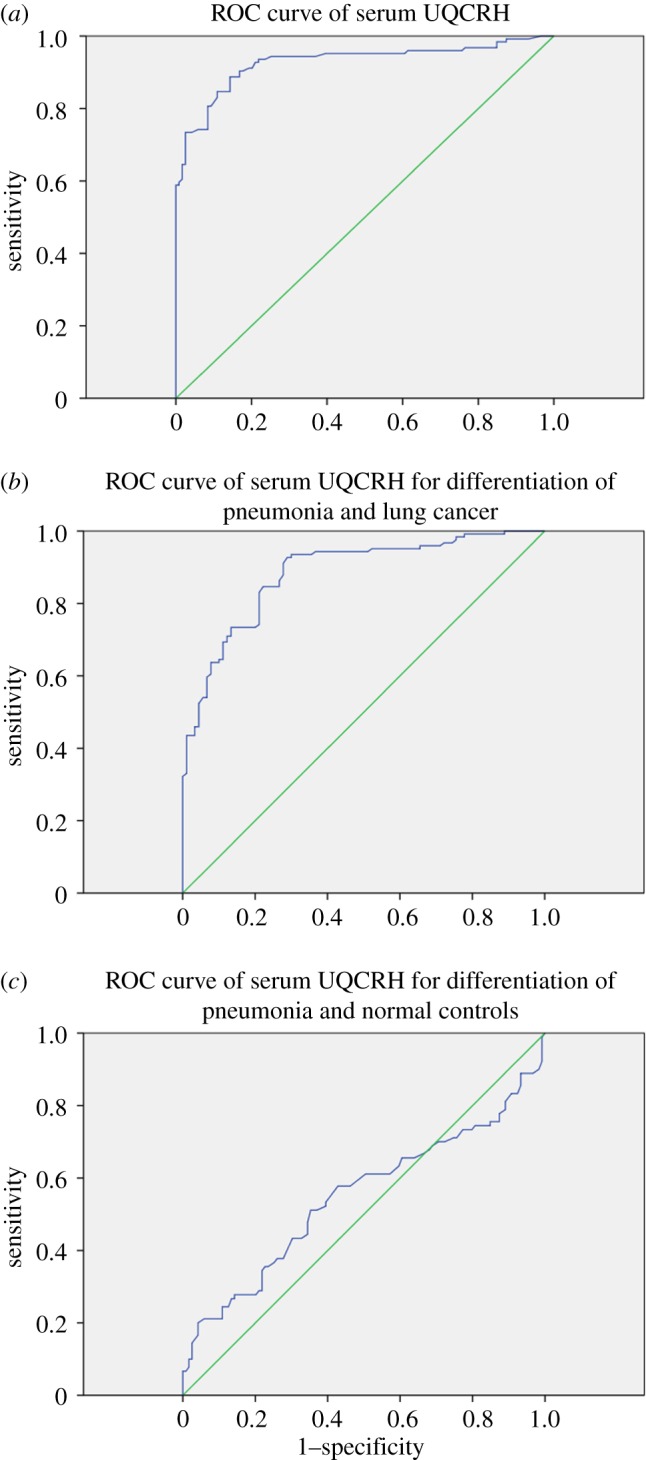
The diagnostic efficiency of serum UQCRH for lung adenocarcinoma. (*a*) The diagnostic efficiency of serum UQCRH for lung adenocarcinoma was studied by the ROC curve method. The area under the ROC curve (AUC) conveys its accuracy for discriminating malignant from normal cases. (*b*) Serum UQCRH can be a good distinction between lung adenocarcinoma and pneumonia. (*c*) Serum UQCRH could not differentiate between pneumonia patients and normal control.

Serum CEA from 65 of the 124 patients with lung adenocarcinoma was higher than its medical decision level (5.0 ng ml^−1^), with a sensitivity of 52.42%. Thus, there were still nearly half of the lung adenocarcinoma patients who would have a missed diagnosis. At the same time, receiver operating characteristic (ROC) curve analysis of serum UQCRH in a group of 124 patients with lung adenocarcinoma and 119 normal controls was used to determine cut-off values. The area under the curve (AUC) for peripheral UQCRH was 0.927 (*p* < 0.0001) for patients with lung adenocarcinoma versus control subjects. A serum UQCRH of 162.65 pg ml^−1^ corresponded to the maximum joint sensitivity and specificity on the ROC curve (88.7% sensitivity and 85.7% specificity, 95% CI 0.892 to 0.962) (figure 4*a*). That is, the sensitivity and specificity of serum UQCRH for diagnosis of lung adenocarcinoma were higher than those of CEA. Next, we used lung adenocarcinoma patients as the experimental group and pneumonia patients as the control group. It was found that serum UQCRH can show a good distinction between lung adenocarcinoma and pneumonia (92.7% sensitivity and 71.1% specificity, 95% CI 0.839 to 0.928) (figure 4*b*), but serum UQCRH could not differentiate between pneumonia patients and normal controls (AUC = 0.549, *p* = 0.230) (figure 4*c*).

## 3. Discussion

Mitochondrial membrane complexes (MMCs) are the key mediators of cellular oxidative phosphorylation, and inhibiting them could lead to cell death. The mitochondrial respiratory chain complexes I, II, III and IV are proteins responsible for electron transport and the associated proton pumping which generates a proton gradient and mitochondrial membrane potential, which is then used (via ATP synthase, complex V) to generate adenosine-5′-triphosphate (ATP), the central energy currency of the cell. Mitochondria also play a key role in apoptosis, through the modulation of membrane potential and the coordinated release of mitochondrial proteins such as cytochrome *c* [12]. One mechanism of mitochondrial targeted anti-cancer drugs relies on their ability to disrupt the energy producing systems of cancer cell mitochondria, leading to increased ROS and activation of the mitochondrial-dependent cell death signalling pathways inside cancer cells [12]. Hinge protein represents subunit 8 of mitochondrial ubiquinol: cytochrome *c* oxidoreductase complex (complex III of the respiratory chain), which has been known for a long time, from biochemical studies, to interact with both cytochrome *c*1 and cytochrome *c*, although its exact function is still unclear. Stable overexpression of hinge protein in a murine promyeloid cell line accelerates apoptosis induced by stressful conditions [13].

**Figure 5.**
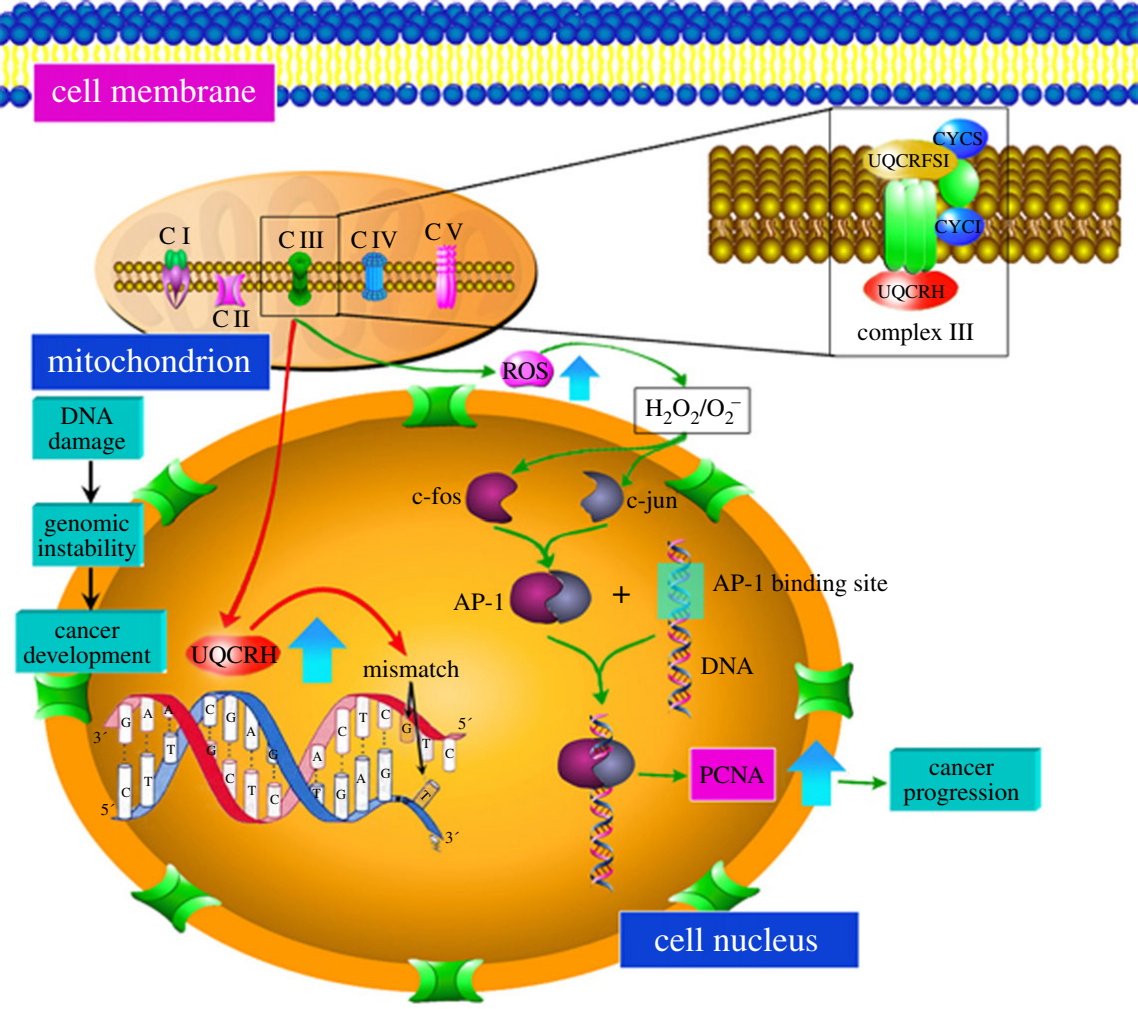
UQCRH in tumour development.

The *UQCRH* gene is located at 1p34.1 and contains five exons. It encodes a hinge protein containing 91 amino acids. UQCRH is distributed in the nucleus and mitochondria, and is mainly involved in mitochondrial oxidative phosphorylation. Based on the outcome of this study and knowledge from the available literature, as shown in the composite scheme in figure 5, we suggest multiple pathways that result in apoptotic cell death or cancer development. As a major subunit of the mitochondrial complex III, UQCRH is responsible for the electron transfer between cytochrome *c* and cytochrome *c*1 during oxidative phosphorylation, and its abnormally high expression may lead to cellular ROS generation, thus contributing to the expression of oncogenes and tumour occurrence and development. This special kind of distribution enables UQCRH to play an important role in functional coordination between mitochondria and the cell nucleus, which is a significant aspect of mitochondrial involvment in cancer [7,9,13]. In aerobic conditions, various cells mainly produce ATP by mitochondrial respiration. In the anoxic condition, healthy cells use anaerobic glycolysis as the main source of energy. However, tumour cells mainly use ‘aerobic glycolysis’ as the way of energy supply [7,10–14], which is related to the oxidative stress of tumour cells [8]. In actively proliferating tumour cells, mutations of the oncogenes contribute to the anomaly of cellular metabolism and protein translation, and result in the rise of ROS. It has been proved that ROS are involved in cell signal transduction and can promote the mitosis of various cells, which leads to tumour occurrence and development [14–16]. In figure 5, on one side H_2_O_2_ and O_2_^−^ induce the expression of c-fos, c-myc and c-jun. Then c-fos and c-jun form AP-1 by dimerization, AP-1 combines with an AP-1 binding site on the *PCNA* gene, and mRNA transcription of *PCNA* targets downstream genes involved in transcriptional regulation, and contributes to carcinogenic effects. On the other side, the high level of UQCRH can cause decrease of the mitochondrial membrane potential in addition to the increase of O_2_^−^ and H_2_O_2_, which results in gene instability or base mismatch, which are initiating factors of tumour development [17]. The higher level of UQCRH in transformed cells corroborates the observation that lung adenocarcinoma is associated with the induction of c-fos, given the fact that the expression of the hinge gene corresponds to the state of cellular differentiation.

UQCRH gene dysfunction might influence tumourigenesis by affecting the superoxide-scavenging activity of the respiratory chain, resembling the hypothesized role of housekeeping genes encoding related mitochondrial proteins, such as succinate dehydrogenases and fumarate hydratase, which act as tumour suppressors in hereditary paragangliomas and uterine fibroids, respectively [18–20].

In this study, UQCRH expression was significantly increased in tumour tissue compared with non-tumourous tissue in lung adenocarcinoma patients. The rate of UQCRH expression in lung adenocarcinoma was as high as 87.10%, while in patients with pulmonary bullae the rate was less than 5%. An elevated UQCRH was significantly associated with sex, smoking history and cancer status. More hearteningly, the serum UQCRH level was significantly higher in lung adenocarcinoma patients than those with pancreatic cancer, though the expression of UQCRH mRNA in pancreatic cancer tissue (as a tumour control) was significantly higher than that in normal tissue. Thus, serum UQCRH is suitable for diagnosis and screening of lung adenocarcinoma. In diagnosing lung adenocarcinoma, the performance of serum UQCRH was better than CEA, with higher sensitivity and specificity. Serum UQCRH could discriminate lung adenocarcinoma patients from other populations without lung cancer with significant sensitivity and specificity. To the best of our knowledge, this is the first study to show increased UQCRH expression in human lung cancer tissue and serum and identifies the usefulness of serum UQCRH as a potential diagnostic biomarker for lung adenocarcinoma. Immunohistochemistry revealed that UQCRH expression was increased in lung adenocarcinoma specimens but not in normal lung tissues. In order to elucidate the role of increased UQCRH expression in the prognosis of patients with lung adenocarcinoma, a prognostic analysis was carried out by using the patients' follow-up data. In the subsequent experiments, the value of serum UQCRH was found to be significantly reduced after operation in patients with lung adenocarcinoma, which illustrates that the serum UQCRH also plays an important role in the surgical and prognostic evaluation of lung adenocarcinoma.

It is generally assumed that after the loss of outer mitochondrial membrane integrity and the release of cytochrome *c* from the mitochondria to the cytosol, the cells are committed to apoptosis [21,22]. It has also been reported that production of ROS contributes to mitochondrial damage that may facilitate the further release of ROS into the cytoplasm [23]. Accordingly, our results demonstrated that UQCRH caused a significant decrease in the mitochondrial membrane potential in A549 cells with UQCRH overexpression. What is the basis of the relationship between increased UQCRH expression and poor prognosis? We believe that increased UQCRH expression may be related to cancer cell proliferation because UQCRH was required in the early stage of DNA replication, along with other hinge protein members (table 1).

In conclusion, our results suggested that increased UQCRH expression in primary lung adenocarcinoma cells plays an important role in the progression of lung adenocarcinoma, and serum UQCRH, obtained non-invasively before surgery, may be one of the simplest measures as a prognostic marker in lung adenocarcinoma. In addition, the present results provide a basis for future investigation into UQCRH overexpression and its clinical implication in other malignancies, which might promote the clinical use of this novel protein.

## 4. Material and methods

### 4.1. Study subjects and specimens

One hundred and twenty-four samples (cancer tissues and serum) of lung adenocarcinoma were obtained from the First Affiliated Hospital of Fujian Medical University between January 2013 and December 2015. All patients met the following criteria: pathological confirmation of lung adenocarcinoma; complete curative resection; no pre-operative treatment; no microscopic residual tumour; no history of transplantation or immunosuppression; no evidence of infection such as pneumonia before surgery; no treatment for concomitant autoimmune diseases with immunosuppressive therapy; no history of acute exacerbation of chronic obstructive pulmonary disease (COPD) or interstitial lung disease within a month before surgery; and availability of laboratory data and follow-up information. Disease stages were based on the 7th edition of the TNM classification of malignant tumours. With an age range of 39 to 68 years, the median age was 54.8 years old. Furthermore, 22 cases of pulmonary bulla (age from 25 to 60 years old, with a median age of 38.9 years) and 53 cases of pathologically confirmed pancreatic cancer (age from 42 to 78, median age 58.2 years) were included, and paired serum samples from 90 cases with pneumonia and 119 healthy individuals served as controls.

### 4.2. Immunohistochemistry analysis

Informed consent was obtained for the use of the specimens, according to the Research Ethics Committee of Fujian Medical University. The sections were incubated with the anti-UQCRH (1 : 150). Sections were washed with deionized water and lightly counterstained with haematoxylin. For each slide, a total of five random images at ×400 magnification were selected and examined with an Olympus microscope system (Tokyo, Japan) to score the staining. The ratio of the number of stained cells to the total number of cells was calculated. Total cell ratio for the staining of UQCRH was ranked into the following three groups, based on the percentage of positive tumour cells: high (+++, greater than 35%), medium (++, 18–35%), low or negative (±, less than or equal to 17%). All of the slides were independently viewed and scored by two pathologists. All of the antibodies were purchased from ABclonal Ltd.

### 4.3. ELISA for serum ubiquinol cytochrome *c* reductase hinge

Serum samples were collected from the patients with lung adenocarcinoma before surgery. At the same time, serum samples were also collected from patients with pulmonary bullae and pneumonia and from healthy control individuals, which served as case control samples. Enzyme-linked immunosorbent assay (ELISA) (R & D Company, USA) was used to detect UQCRH.

### 4.4. Evaluation of the sensitivity and specificity of serum ubiquinol cytochrome *c* reductase hinge as marker for lung adenocarcinoma

The ROC curves were analysed to determine the optimal cut-off value and diagnostic sensitivity and specificity; serum UQCRH was compared with CEA.

### 4.5. Detection of ubiquinol cytochrome *c* reductase hinge mRNA in lung cancer tissues

RNA was extracted from lung cancer tissue and normal lung tissue away from the cancer using TRI Reagent^®^ (Applied Biosystems, Foster City, CA, USA). UQCRH forward 5′-AGGGACCATTGCGTGGCC-3′ and reverse 5′-AGCTACCAGCCTAAGCCAAA-3′ were used as upstream and downstream primers. RT-qPCR was performed under the following conditions: 95°C 15 s, 67°C 1 min for 35 cycles with an initial denaturation at 95°C for 10 min. The same method was used for quantitative detection of UQCRH mRNA in normal tissues of pancreas and pancreatic cancer tissue.

### 4.6. Mitochondrial membrane potential (△*Ψ*m) analysis

The A549 human lung adenocarcinoma cells were obtained from ATCC (American Type Culture Collection; Rockville, MD), grown in RPMI 1640 containing 10% heat-inactivated fetal bovine serum (FBS) and maintained at 37°C in a humidified incubator containing 5% CO_2_. Exponentially growing cells were exposed to drugs for the indicated time periods. All culture reagents were purchased from Gibco/BRL. A549 cells (with overexpression or normal expression of UQCRH) were seeded in six-well plates at a density of 1.5 × 10^5^ cells well^−1^. Cells were incubated in medium containing 100 µg ml^−1^ of JC-1 (5,5′,6,6′-tetrachloro-1,1′,3,3′-tetraethylbenzimidazolylcarbocyanine iodide) according to the manufacturer's instructions for 24 h. JC-1 Mitochondrial Membrane Potential Detection Kit from Cell Technology (Mountain View, CA, USA) was used. The JC-1 dye bearing a delocalized positive charge enters the mitochondrial matrix due to the negative charge established by the intact △*Ψ*m. In healthy cells, JC-1 dye stains the mitochondria red. In apoptotic cells, JC-1 dye accumulates in the cytoplasm in monomeric form (green fluorescence). Stock solution of JC-1 (100 µg ml^−1^) was prepared in DMSO and freshly diluted with the assay buffer supplied by the manufacturer. △*Ψ*m in A549 cells with normal expression and after UQCRH overexpression was detected by inverted fluorescence microscope. The fluorescent probe Mitotracker-Red was excited by blue light and red light, reflecting the mitochondrial membrane potential.

### 4.7. Western blot analyses

UQCRH and c-fos in A549 cells with UQCRH overexpression or normal expression was detected by western blot. That is, proteins were separated on 4 to 12% Tris-glycine gels and transferred to nitrocellulose membranes. Complete and uniform transfer was confirmed by Ponceau S staining. Membranes were probed with antibodies directed against c-fos (ABclonal), UQCRH (ABclonal) and GAPDH (ABclonal).

### 4.8. Statistical analysis

The relationship between clinicopathological parameters and UQCRH was analysed by *χ*^2^ test. SPSS 14.0 was used to carry out data processing; the statistical indexes were normal and the homogeneity of variance was tested, and the difference was statistically significant with *p* < 0.05.

## Data accessibility

The datasets supporting this article have been uploaded as part of the electronic supplementary material.
